# Intervertebral Disc Biology: Genetic Basis of Disc Degeneration

**DOI:** 10.1007/s40610-018-0101-2

**Published:** 2018-08-03

**Authors:** Sabrina Munir, Marinko Rade, Juhani H. Määttä, Maxim B. Freidin, Frances M. K. Williams

**Affiliations:** 1 Department of Twin Research and Genetic Epidemiology, School of Life Course Sciences, King’s College London, London, UK; 2 Department of Physical and Rehabilitation Medicine, Kuopio University Hospital, Kuopio, Finland; 3 Faculty of Medicine, Orthopaedic and Rehabilitation Hospital “Prim. dr.Martin Horvat”, Josip Juraj Strossmayer University of Osijek, Rovinj, Croatia; 4 Medical Research Center Oulu, Oulu University Hospital and University of Oulu, Oulu, Finland

**Keywords:** Intervertebral disc degeneration (IDD), Low back pain (LBP), Candidate genes, Heritability, GWAS, Spine

## Abstract

**Purpose of Review:**

This review aims to highlight recent advances in understanding the genetic basis of intervertebral disc degeneration (IDD).

**Recent Findings:**

It has been known for some time that IDD is highly heritable. Recent studies, and in particular the availability of agnostic techniques such as genome-wide association studies, have identified new variants in a variety of genes which contribute to the risk of IDD and to back pain.

**Summary:**

A variety of genetic variants are involved in IDD. Some are shared with variants predisposing to back pain, but few have been identified reliably in either phenotype. Further research is required to explain fully the high heritability and how the genetic variants influence cell biology to lead to IDD.

## Introduction

Low back pain (LBP) is a highly prevalent disabling musculoskeletal condition and a leading cause of activity limitation and work absence [1••, 2]. The lifetime prevalence of LBP has been reported as over 80% and the global age-standardised point prevalence of LBP estimated to be 9.4% (95% CI 9.0–9.8) [1••, 3]. LBP associated long-term sick leave or unemployment ensues an enormous economic burden [4]. Combined, the high prevalence of LBP and burden of healthcare and socioeconomic costs make it an important public health issue [5]. A major cause of LBP is intervertebral disc degeneration (IDD) [6••]. IDD is found to be more prevalent among subjects with LBP than without [7]. The aetiology of IDD is complex with both environmental and genetic influences, but over the last few decades, it has become clear that genetic influences predominate [8, 9]. However, the precise mechanisms underlying IDD remain unclear. This review will focus on the recent advances in understanding the genetic basis of IDD, highlighting the results from research in the field over the last 5 years.

## Structure and Function of Intervertebral Disc

Intervertebral discs (IVDs) are located between vertebral bodies [10]. Their primary role is mechanical, as they are designed to transmit forces through the spine and minimise shock. The IVD in adult humans is an avascular, complex structure composed of a central gelatinous nucleus pulposus (NP) surrounded by an outer ring of fibrous cartilage named the annulus fibrosis (AF).

The AF comprises a series of 15 to 25 concentric rings or lamellae containing collagen fibres aligned in parallel and orientated at a 60-degree angle to the vertical axis, which alternate directional between in successive layers, providing tensile strength and the ability to withstand forces applied from any direction.

Unlike the dense rigid AF encompassing it, the NP has a gel-like consistency [11]. The NP is made of collagen and elastin fibres embedded in a hydrated proteoglycan containing gel, which is able to resist compressive loads and to deform under stress. The distinction between the NP and the inner layer of AF becomes less apparent and disappears early in life [12]. The region of transition between the two types of tissue is often referred to as the transition zone.

The IVD is sandwiched between two vertebral endplates of adjacent vertebral bodies. The endplates are bilayers of hyaline cartilage and bone [11, 13]. The cartilaginous endplate is contiguous only with the NP and the inner AF. Collagen fibres of the outer AF anchor directly into the bone of the apophyseal ring facilitating the transmission of tensile loads [12]. The vertebral endplate acts as an interface between the IVD and adjacent vertebral body and has an important role in disc nutrition. Metabolites and nutrients move down the diffusion gradients established by the metabolic demands of the disc cells [14]. Trans-endplate diffusion is enhanced by forced convection, as IVDs are cyclically mechanically pressurised and unloaded [15].

### Aetiology of IDD

The aetiology of IDD is complex and multifactorial with ageing, smoking, injuries and genetic factors all playing a role [10]. Degenerative change to the disc due to ageing and pathological changes is a contentious area because the two are difficult to differentiate [16]. Structural defects in the endplate or IVD affect detrimentally the mechanical environment leading to degenerative change in the disc [17]. Endplate damage is thought to induce disc degeneration through both mechanical and nutritional factors. Damage to the endplate reduces pressure in NP provoking nutritional disturbances in the disc [18]. Additionally, calcification of the endplate may disturb the nutrition of the disc [19]. Progressive decrease in disc nutrient supply and alterations in the ECM composition occur, resulting in weakening of tissue strength and changes in cell metabolism, leading to further degeneration [20]. A recent study performed on a large population-based sample confirmed that endplate defects are strongly associated with IDD [21•]. How these observations tie in with the known genetic influence on IDD is yet to be explained.

Inflammation is believed to play a role in the development of IDD [22]. However, it is still unclear whether inflammation is the trigger or a consequence of IDD. Both local inflammation through the upregulation of proinflammatory cytokines and chemokines as well as systemic inflammation have been linked with IDD and disc-related LBP.

IDD is a complex disease with genetic and environmental influences. IDD can be sporadic or familial, as individuals may inherit a set of genes increasing their risk of developing IDD. In this review, we are considering sporadic forms of IDD.

### Heritability of IDD and Approaches to Reveal Underlying Genetic Factors

The importance of genetic variation as a risk factor for IDD was established through the use of twin studies. The heritability of IDD was initially investigated using 115 male monozygotic twin pairs from the population-based Finnish Twin Cohort. Through multi-variate association testing, it was found that familial aggregation explained 61 and 34% in the upper and lower regions of the lumbar spine, respectively, of lumbar disc degeneration (LDD) scores [8]. This was followed by a classical twin study heritability estimates of degenerate disc phenotypes (including disc signal, bulging and height narrowing) ranging between 29 and 54% depending on the lumbar level [23]. Sambrook et al. (1999) conducted the first classical twin study comparing intraclass concordance of monozygotic and dizygotic twin pairs in the TwinsUK cohort which is predominantly female [9]. They reported heritability estimates of 76% in the lumbar spine and 73% in the cervical spine which are surprisingly high for a condition which was previously considered occupational (environmental) in nature. A later study using TwinsUK identified a significant genetic correlation between the LBP and LDD measurements and suggested that 11–13% of the genetic effects are shared by both LDD and LBP [24].

Once a condition has shown to be heritable, the next step is to identify the genetic variants involved. Genetic markers identified may confer a risk or protective effect in the development of IDD. The main approaches used in the 1990s were linkage studies of related individuals and candidate gene association studies. In linkage studies, co-segregation of genetic markers and phenotypes within pedigrees is used to identify regions of the genome likely to harbour genes associated with the trait. In comparison, the candidate gene approach is used to assess association between a phenotype and genetic variation within a gene of interest considered to be a plausible candidate based on knowledge regarding its function. Candidate gene approaches have been a mainstay in the study of association between a gene of interest and a phenotype. This is primarily because they are relatively cheap and quick to perform. As this approach involves the selection of genes related to the disease or phenotype, a priori of knowledge is required. However, limited knowledge may restrict the ability to identify associated genes.

Genome-wide association studies (GWAS) became available from 2007 thanks to the International HapMap project. Such studies provide an agnostic way of identifying common variants (usually single-nucleotide polymorphisms, SNPs) associated with a phenotype. In a GWAS, common variants across the whole genome are used to tag a region of linkage disequilibrium (LD), which are then tested for association with a trait. Those tag SNPs significantly associated with the trait or phenotype lie in an LD block containing a causal SNP. Such studies require well-defined case and control groups with a sufficient sample size to achieve adequate statistical power. Furthermore, often variants identified are of small effect size and rare variants associated with the phenotype are not identified, thus providing explanation for only a small proportion of heritability usually explained by the GWAS-identified variants associated with the phenotype.

A number of excellent reviews have been published focussing largely on the extensive candidate gene literature [25•, 26•]. The genetic variants and studies which will be discussed have been selected to highlight findings reported in the past 5 years with a particular focus on those found associated using agnostic methods.

## Candidate Genes in IDD

### Thrombospondin

Thrombospondin proteins (THBSs) are a class of glycoproteins with a variety of different functions in the extracellular matrix (ECM) [27]. THBSs bind to collagen and tissue and participate in cell-to-cell and cell-to-matrix interactions during tissue development and repair. THBSs regulate the level of matrix metalloproteinase-2 (MMP-2) and MMP-9, which are thought to play an important role in IDD [27]. Some functional polymorphisms in *THBS2* have been found to be associated with lumbar disc herniation and lumbar spinal stenosis in the Japanese and Korean population [28]. More recently, two SNPs (rs6422747 and rs6422748) in the *THBS2* gene were associated with susceptibility to IDD but not severity of IDD in a Chinese Han population, indicating that *THBS2* gene polymorphisms might be risk factors for IDD [29].

### Vitamin D Receptor

Vitamin D receptor (*VDR*) gene is one of the most extensively studied candidate genes in IDD. Studies that have investigated the association between *VDR* gene polymorphisms and IDD have yielded contradictory results. Polymorphisms in *VDR* including TaqI (rs731236), FokI (rs2228570) and ApaI (rs7975232) have all been reported to be associated with IDD; however, results from two recent meta-analyses have concluded that there is no association between *VDR FokI* and *TaqI* polymorphisms and IDD [30•, 31•]. Nevertheless, in the meta-analysis reported by Chen et al. (2017), subgroup analysis by ethnicity found that the *FokI* mutation was associated with a significantly lower risk for IDD among Caucasians. The ethnic specific protective effects of the *FokI* polymorphism were supported by evidence from a meta-analysis reported by Pabalan et al. (2017) [32•]. However, the protective effect of the *FokI* polymorphism was not replicated in the meta-analysis reported by Jiang et al. (2017) in Caucasian and Asian populations. Furthermore, Pabalan et al. provided evidence that the *ApaI* polymorphism in *VDR* reduces the risk of IDD. Discrepancies in the findings of these meta-analyses highlight that further large-scale and well-designed studies are required to clarify the possible role of *VDR* polymorphisms in IDD and to provide a definitive conclusion but it would seem likely that if there is an effect, it is small and specific to ethnicity.

### Collagens

As described above, several types of collagen form an essential part of the IVD. Variants in the genes encoding type I, IX and XI collagens have been identified [10]. In more recent years, the role of the genes encoding type II collagen has been explored. Collagen type II, alpha 1 (*COL2A1*) encodes the alpha-1 chain of type II collagen, a major collagen in cartilage. A previous study indicated that rs2276454 and rs2070739 variants were predisposing factors of IDD [33]. The variants rs2276454 and rs1793953 have been found to be associated with an increased and decreased risk of developing IDD, respectively [34], thus confirming the risk associated with the variant rs2276454.

### Aggrecan

Aggrecan, encoded by the gene *ACAN*, is a proteoglycan with many glycosaminoglycan side chains located in the IVD matrix and vertebral endplate [35]. A variable number of tandem repeat (VNTR) polymorphism in the *ACAN* gene were found associated with IDD. The VNTRs range from 13 to 33 nucleotides, with the most common number being 26, 27 or 28 repeats [36]. Results from meta-analysis identified a 1.54-fold increased risk of lumbar disc degeneration (LDD) for the shorter allele carriers compared with the normal and longer alleles [35]. Subgroup analysis revealed significant increased risk of LDD among Asians with shorter alleles. The results from this study highlighted that shorter VNTRs are associated with an increased risk of IDD, especially among those of Asian descent. There is also evidence that *COL2A1* and ACAN genetic polymorphisms may be correlated with the risk and features of IDD in a Chinese Han population [34].

### Aggrecanase

It has been shown that the aggrecanases may contribute to the changes occurring in the ECM during IDD. Aggrecanase-1 (a disintegrin and metalloprotease with thrombospondin motifs-4, *ADAMTS4*) and aggrecanase-2 (*ADAMTS5*) are two aggrecanases thought to play a role in IDD. The SNP rs4233367 in the exon of *ADAMTS4* has been found to be associated with LDD [37]. The T allele at this SNP conferred a lower risk of LDD with an OR of 0.69 and TT genotype is at almost one-fifth of the risk compared to CC genotype. In the gene, *ADAMTS-5*, the SNP rs162509 was found to be associated with IDD [38]. Furthermore, the A alleles of the rs151058, rs229052 and 162502 intronic variants of the *ADAMTS5* gene were all found associated with LDD [39]. In mouse models, *ADAMTS5* deficiency was found to be protective against chronic tobacco smoking-induced IDD, providing further evidence that aggrecanases may have a role in mediating an environmental risk factor for IDD [40].

### Interleukins

Interleukins (IL1α, IL1β and IL6) are pro-inflammatory cytokines [41]. IL-1 is normally expressed in the IVD and is responsible for indirectly degrading ECM components through the production of degradative enzymes, upregulation of other cytokines and preventing the production of ECM components. Meta-analysis suggested that the IL-1alpha (+889C/T) polymorphism is significantly associated with risk of IDD, especially in Caucasian populations [42•]. The SNP rs1800587 in *IL1A* has been associated with both early LDD in young girls and Modic change (MC), an MRI trait associated with IDD [43, 44]. More recently in a case/control study of 332 subjects drawn from the Indian population with highly specific phenotypes for disc degeneration, the variant rs1800587 was not found to be associated with any of the three highly specific markers for IDD, namely disc degeneration by Pfirrmann grading, endplate damage evaluated by total endplate damage score and annular tears evaluated by disc herniations and hyperintense zones. In another study, the rs2856836, rs1304037, rs17561 and rs1800587 variants of the *IL1A* gene were associated with the severity of LDD and MC [45]. Of note, the rs17561 variant of *IL1A* was predicted as pathogenic by the PolyPhen prediction tool [45].

IL-6 is thought to have an important role in lumbar disc herniation [46]. Polymorphisms in *IL6* have been reported to be significantly associated with IDD. A 15T/A substitution in exon 5 of *IL6* was associated with a 4.4-fold increased risk of IDD in patients with AA or AT genotypes compared to the TT genotype [47]. In a sample of Danish girls, the SNP rs1800796 was found to confer a very high—6.7-fold—increased risk of developing IDD in girls carrying the C allele compared to those without the allele [43]. This association was not observed in boys. In 2012, a study reported two different protective polymorphisms (rs1800797 and rs180079) in *IL6* that were found to be only associated with IDD in adolescent boys [46]. In a recent meta-analysis, rs1800797 (genotype CC) and rs1800795 (genotype CC) were found to confer increased and decreased risk of developing IDD, respectively [48•]. A recent study found no association of *IL6* with severity of lumbar disc herniation in an Indian patient sample [45]. However, in a Han Chinese population, it was found that the relative risk of developing lumbar disc herniation with the *IL-6*-572 G genotype GG and CG genotypes were also high—4.48- and 1.55-fold—higher than the CC genotype [49]. Providing evidence shows that genetic variants in the promoter regions of the *IL-6* are associated with lumbar disc herniation. Results regarding the role of IL-6 suggest that polymorphisms may be associated with an increased risk of IDD or may confer protection; however, these may be gender or population specific.

### Matrix Metalloproteinases

The degradation of the disc matrix by matrix metalloproteinases (MMPs) is thought to play an important role in IDD. In particular, recent work has explored the role of *MMP3* and *MMP9* in IDD. In a North Iranian population, the homozygous variant (CC) of the polymorphism rs632478 in *MMP3* resulted in 5-fold significant increased risk for disc degeneration relative to the AA variant [50]. Also, Takahashi et al. reported that the *MMP3* 5a5a and 5a6a genotype was associated with a significantly larger number of degenerative discs than the 6a6a in the elderly [51•]. Meta-analysis found that MMP-9 rs17576 increased significantly the risk of disc degeneration [48•]. The role of MMPs has also been explored in relation to MC. In a sample of Indian origin, the rs17099008 SNP of *MMP20* was found to be significantly associated with MC (*p* = 0.03) [52]. These results highlight that MMPs may have a role in IDD and MC, a feature of IDD.

### CILP

Recently, a single-nucleotide polymorphism rs2073711 of the cartilage intermediate layer protein (CILP) gene has been associated with IDD [53•]. Meta-analysis revealed that the polymorphism was significantly associated with IVD risk (odds ratio (OR) = 1.36, 95% CI: 1.18–1.55, *P* < 0.001). Further subgroup analysis found similar ORs for both Asian and European populations. A recent study identified a significant association (*p* < 0.01) of three SNPs of *CILP* and disc bulge, a feature of IDD [54]. Further analysis is required to identify whether variants in CILP are associated with IDD as a whole, or specific features of IDD.

### TRAIL

Tumour necrosis factor-related apoptosis-inducing ligands (TRAILs) are transmembrane proteins that belong to tumour necrosis factor ligands [55]. Studies examining the association between *TRAIL* and IDD have previously produced inconsistent results. Newer studies have shed light on the association between *TRAIL* and LDD.

Significant association between *TRAIL* 1525/1595 polymorphisms and the risk of LDD have been reported in Han Chinese population [56–58]. All three studies reporting *TRAIL* 1595C/T gene polymorphisms were included in meta-analysis and a significant relationship between 1595C/T polymorphisms and increased IDD risk was found (OR = 2.18, 95% CI 1.45 to 3.27, *P* < 0.0001) [55]. Patients with lower grade IDD had higher frequency of the 1595TT genotype and 1595T allele. These results highlight that *TRAIL* 1595C/T polymorphisms in fact have a role in IDD. Death receptor-4 (DR4) and death receptor-5 (DR5) are both receptors that bind to TRAIL and induce apoptosis within the target cell [36]. The C626G polymorphism of *DR4* gene with CG and GG genotypes was associated with the risk and severity of LDD compared to CC genotypes in a Han Chinese population [59]. Furthermore, the G allele was associated with higher degenerative grades of LDD compared with the CC genotype and the C allele. The associations of *TRAIL* and *DR4* have only been investigated in Han Chinese population. Studies using other ethnic populations are required to see if these results are ethnic specific.

### GDF5

Variants in the gene *GDF5* were initially thought to predispose individuals to osteoarthritis (OA), therefore believed to also be a good candidate for LDD. The same risk allele of the SNP rs143383 in *GDF5* as in knee and hip OA was found to be associated with LDD in five predominantly female cohorts of Northern European descent (OR = 1.72; 95% CI = 1.15–2.57; *p* = 0.008) [60•]. A more recent meta-analysis demonstrated a significant association between the rs143383 polymorphism and the susceptibility to LDD, with the T allele conferring risk and the C allele protection [61]. In sub-group analysis, significant association was observed in Caucasian and Asian subgroups.

### SKT

The human *SKT* gene (*KIAA1217*) was believed to be a good candidate for a lumbar disc herniation (LDH) susceptibility because *SKT* is expressed in NP of IVDs. A significant association with the SKT variant rs16924573 was found in two independent Japanese case-control populations and replicated Finnish case-control population [62]. Meta-analysis using more than 2200 Japanese and Finnish subjects confirmed the association between the SNP rs16924573 and LDH (OR = 1.34; 95% CI = 1.14–1.58, *p* = 0.00040).

## Genetic Variants Revealed by Genome-Wide Association Studies

### PARK2

The first GWAS investigating disc degeneration was reported in 2012 [63]. A genome-wide association meta-analysis was conducted using 4683 individuals of European ancestry and four SNPs associated with LDD were identified. The most significant SNP was within an intronic region of the *PARK2* gene, which encodes parkin, a component of a multiprotein E3 ubiquitin protein ligase complex. Differential methylation at a CpG island of the *PARK2* promoter was observed in a small subset of subjects (*p* = 0.006), suggesting that methylation of the *PARK2* promoter may influence degeneration of the IVD. These results in Northern Europeans, although intriguing and arising from a hypothesis-free method, have yet to be replicated.

### CHST3

Evidence from a combined genome-wide linkage analysis of families with early-onset LDD and a large GWAS meta-analysis using multi-ethnic population samples identified a novel candidate gene *CHST3* encoding carbohydrate sulfotransferase 3 [64•]. Genome-wide significance was reached for the SNP rs4148941 in association with LDD. Additionally, expression of *CHST3* mRNA was significantly reduced in the IVD cells of human subjects carrying the A allele of rs4148941. Together, this evidence suggests that the gene *CHST3* has a role in LDD.

### Genes for Sciatica

The first GWAS and meta-analysis of sciatica was performed using two Finish cohorts, which identified two novel associated loci, *NFIB* and *MYO5A* [65•]. The most significant association in the meta-analysis, the SNP rs71321981 located in the regulatory region of the transcription factor *NFIB*, was replicated in an independent Finnish population sample (*p* = 0.04). However, the association with the *MYO5A* gene could not be replicated. The SNP rs115488695 representing *HLA-DRB5* did not quite reach genome-wide significance (*p* = 3.58 × 10^−7^); however, SNPs (rs2187689, rs7767277) in the nearby gene *TAP1* were associated with LDD in the meta-analysis of Northern European individuals reported above [63].

A GWAS on 4748 cases of sciatica with proven herniated lumbar disc who underwent microdiscectomy (LDHsurg) and 282,590 population controls identified 37 highly correlated markers associating with LDHsurg at 8q24.21, with the lead SNP rs6651255 (OR = 0.81; *p* = 5.6 × 10^−12^) [66]. Its effect was found to be stronger among younger patients than older (*p* = 1.8 × 10^−3^). The SNP and variants in strong LD are eQTLs affecting expression of nearby genes *GSDMC* and *CCDC26*. A Mendelian randomisation analysis was performed to establish a possible causative role of height on the risk of herniated disc and showed no such effect using the associated SNPs as instrumental variable. It has been suggested that the effect of rs6651255 on risk of LDHsurg is driven by the severity and persistence of associated sciatica, rather than by the morphology associated with herniated lumbar discs.

### Genes for Back Pain

Large GWAS meta-analysis for chronic back pain has recently been conducted based on the data from UK Biobank and Cohorts for Heart and Ageing Research in Genomic Epidemiology (CHARGE) consortium cohorts which included a total of 158,000 participants [67•].The study identified and replicated a novel locus for chronic back pain tagged by the intronic variant rs12310519 in *SOX5* gene (OR 1.08, *p* = 7.2 × 10^−10^). Two other loci reached genome-wide significance in a two-stage meta-analysis: one tagged by an intergenic variant, rs7833174, located between *CCDC26* and *GSDMC* genes (OR 1.05, *p* = 4.4 × 10^−13^), and another tagged by an intronic variant, rs4384683, in *DCC* genes (OR 0.97, *p* = 2.4 × 10^−10^). The finding of the same genetic variants associated with a rather vague back pain questionnaire and in a highly specific group of patients undergoing discectomy for MR-proven disc prolapse is intriguing and promising. Bioinformatic work up will provide assistance in localising the genetic variant providing the signal. It gives hope to further work using many different approaches, not just the highly phenotyped clinical cases. All GWAS studies, however, require very large samples to be successful and this recent work has highlighted the extreme polygenicity associated with the back-pain phenotype. Further work is under way using the UK Biobank sample of 500,000 individuals [68].

## Conclusion

While the high heritability of sporadic LDD was recognised in the 1990s, progress to identify the variants involved has been slow. This reflects a number of factors, such as the difficulty of studying the IVD itself to identify suitable candidate genes, and the recognition that the candidate gene approach itself is often flawed. This is because the selection of suitable controls often leads to occult population stratification which leads to bias in the results. The literature is likely to contain many false positive associations which will not withstand the rigour of the agnostic methods such as GWAS. The GWAS, while a powerful way to identify novel variants, requires large samples of individuals with standardised phenotyping and a collaborative approach to data sharing around the world. This is now beginning to happen, with the EU FP7 projects Genodisc and PainOmics, and new genes are being implicated in LDD which will shed light on the pathogenesis of the condition and may reveal novel therapeutic targets. Other approaches may also be helpful such as exome sequencing within families which has been successful in identifying a novel variant in *IGFBP6* [69]. Studies investigating the role of therapeutic protein injections, stem cell injections and gene therapy show promising results [20]. However, deepening our knowledge of IDD and identification of the most important candidate genes is required to aid the development of successful biologic and gene IVD therapies in the future, in an effort to treat or delay onset in patients at high risk of IDD.
